# SARS-CoV-2 Spike Proteins and Cell–Cell Communication Induce P-Selectin and Markers of Endothelial Injury, NETosis, and Inflammation in Human Lung Microvascular Endothelial Cells and Neutrophils: Implications for the Pathogenesis of COVID-19 Coagulopathy

**DOI:** 10.3390/ijms241612585

**Published:** 2023-08-09

**Authors:** Biju Bhargavan, Georgette D. Kanmogne

**Affiliations:** Department of Pharmacology and Experimental Neuroscience, College of Medicine, University of Nebraska Medical Center, Omaha, NE 68198-5800, USA; bbhargavan@unmc.edu

**Keywords:** SARS-CoV-2 spike proteins, human lung endothelial cells, neutrophils, P-selectin, von willebrand factor, IL-6, citrullinated histone H3, neutrophils extracellular traps, TFPI, DTNB, thrombomodulin

## Abstract

COVID-19 progression often involves severe lung injury, inflammation, coagulopathy, and leukocyte infiltration into pulmonary tissues. The pathogenesis of these complications is unknown. Because vascular endothelium and neutrophils express angiotensin-converting enzyme-2 and spike (S)-proteins, which are present in bodily fluids and tissues of SARS-CoV-2-infected patients, we investigated the effect of S-proteins and cell–cell communication on human lung microvascular endothelial cells and neutrophils expression of P-selectin, markers of coagulopathy, NETosis, and inflammation. Exposure of endothelial cells or neutrophils to S-proteins and endothelial–neutrophils co-culture induced P-selectin transcription and expression, significantly increased expression/secretion of IL-6, von Willebrand factor (vWF, pro-coagulant), and citrullinated histone H3 (cit-H3, NETosis marker). Compared to the SARS-CoV-2 Wuhan variant, Delta variant S-proteins induced 1.4–15-fold higher P-selectin and higher IL-6 and vWF. Recombinant tissue factor pathway inhibitor (rTFPI), 5,5′-dithio-bis-(2-nitrobenzoic acid) (thiol blocker), and thrombomodulin (anticoagulant) blocked S-protein-induced vWF, IL-6, and cit-H3. This suggests that following SARS-CoV-2 contact with the pulmonary endothelium or neutrophils and endothelial–neutrophil interactions, S-proteins increase adhesion molecules, induce endothelial injury, inflammation, NETosis and coagulopathy via the tissue factor pathway, mechanisms involving functional thiol groups, and/or the fibrinolysis system. Using rTFPI, effectors of the fibrinolysis system and/or thiol-based drugs could be viable therapeutic strategies against SARS-CoV-2-induced endothelial injury, inflammation, NETosis, and coagulopathy.

## 1. Introduction

Severe acute respiratory syndrome coronavirus-2 (SARS-CoV-2), the causative agent of coronavirus disease 2019 (COVID-19), has so far infected over 686 million people worldwide, resulting in over 6.88 million deaths and counting [1,2,3]. Although SARS-CoV2-induced immunopathology can affect several organs, postmortem examination shows that for most COVID-19 patients, the primary cause of death was acute lung injury associated with the presence of virions and spike (S) proteins in lung blood vessels, endothelial injury, increases in leukocyte infiltration in lung tissues, circulating prothrombotic factors, inflammation, and thrombosis [4,5,6,7]. Endothelial injury is also associated with the release of von Willebrand factor (vWF) from endothelial granules, upregulation of adhesion molecules, increased neutrophil activation, adhesion and transmigration into vascular walls [8,9]. The pathogenesis of these pulmonary complications in COVID-19 patients is unknown.

Coronaviruses enter and infect target cells by binding their S-protein to cellular angiotensin-converting enzyme-2 (ACE2) [10,11], and human neutrophils [12,13] and endothelial cells [14,15,16] express ACE2. Because SARS-CoV-2 and its S-proteins are present in tissues and bodily fluids of infected patients and COVID-19 pathology includes endotheliopathy and leukocyte infiltration into the lungs [7,17,18], it is important to determine whether viral S-proteins directly contribute to these lung pathologies and whether leukocyte interactions with the vascular endothelium influence SARS-CoV-2-induced pathologies. In the present study, we investigate the direct and indirect effects of S-protein exposure on the expression and secretion of adhesion molecules, markers of endothelial injury, and inflammation. Because increased levels of neutrophil extracellular traps (NETs) are associated with COVID-19 pathology and disease severity [19,20,21,22], we also investigated the direct and indirect effects of S-protein exposure on markers of NET activation and release (NETosis). We demonstrate that exposure of primary human lung microvascular endothelial cells (HLMEC) or neutrophils to S-proteins and endothelial–neutrophil interactions induced transcription and expression of P-selectin and significantly increased the expression and secretion of vWF, interleukin (IL)-6, and citrullinated histone H3 (cit-H3), a marker of NETosis. A trend toward higher P-selectin and vWF levels and significantly higher IL-6 levels was observed with SARS-CoV-2 Delta variant S-proteins (SD) compared to Wuhan variant S-proteins (SW). Recombinant tissue factor pathway inhibitor (rTFPI; the primary physiological inhibitor of the extrinsic pathway of blood coagulation [23,24,25,26]), as well as 5,5′-dithio-bis-(2-nitrobenzoic acid) (DTNB; a thiol blocker) and thrombomodulin (TM; a high affinity thrombin receptor [27]), blocked S-protein-induced expression of vWF, IL-6, and cit-H3.

These data suggest that when the lung endothelium or neutrophils are exposed to SARS-CoV-2, viral S-proteins increase adhesion molecules and induce endothelial injury, inflammation, and NETosis via the TF pathway and mechanisms involving functional thiol groups and the fibrinolysis system. Furthermore, any of these two cell populations exposed to SARS-CoV-2 or viral S-proteins can induce injury, inflammation, and NETosis in non-exposed neighboring cells. These findings suggest that viable therapeutic strategies against SARS-CoV-2-induced cellular injury, NETosis, and inflammation could include rTFPI, effectors of the fibrinolysis system, and/or thiol-based drugs.

## 2. Results

### 2.1. Exposure of HLMEC or Neutrophils to S-Proteins and Endothelial–Neutrophil Interactions Increased P-Selectin Transcription

**Figure 1:**
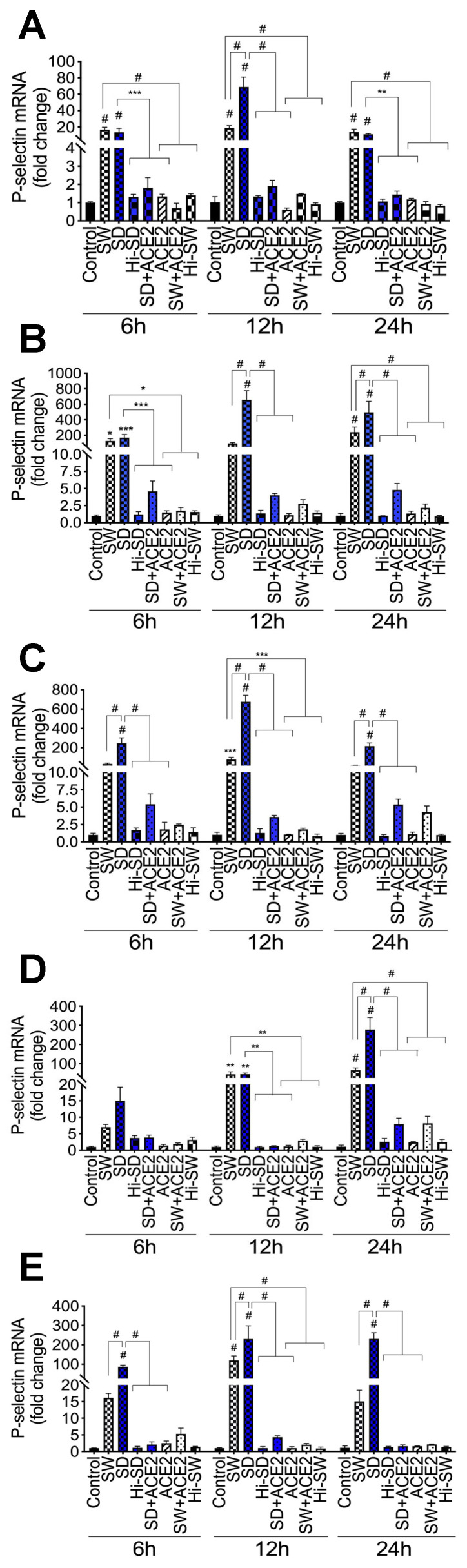
S-proteins and endothelial–neutrophil interactions induce P-selectin transcriptional upregulation in HLMEC and neutrophils. (**A**): HLMEC treated (for 6–24 h) with 1 nM S-protein Wuhan (SW) or Delta (SD) variants. (**B**,**C**): HLMEC were treated (6 h) with SW or SD, washed, and co-cultured (6–24 h) with neutrophils. (**D**,**E**): neutrophils treated (6 h) with SW or SD, washed, and co-cultured (6–24 h) with HLMEC. P-selectin mRNA in endothelial cells (**A**,**B**,**D**) and neutrophils (**C**,**E**) was quantified by real-time PCR. Data presented as mean ± standard deviation. Control: untreated cells; ACE2: cells treated with recombinant human (rh) ACE2 (1 µg/mL). Hi: cells treated with heat-inactivated S-proteins. * *p* < 0.015; ** *p* < 0.007; *** *p* < 0.0007; # *p* < 0.0001.

Compared to controls (untreated cells, cells treated with heat-inactivated S-proteins, or cells pretreated with recombinant human (rh) ACE2), exposure (6–24 h) of HLMEC to SW or SD increased P-selectin mRNA by 12- to 20-fold and 10- to 67-fold, respectively; with the largest increase (51.7- to 67-fold) observed at 12 h (Figure 1A). Co-culture of SW- or SD-treated HLMEC with neutrophils increased P-selectin mRNA in HLMEC by 64.7- to 258-fold and 138- to 650-fold, respectively (Figure 1B), and increased P-selectin mRNA in neutrophils by 17- to 92-fold and 148- to 652-fold, respectively (Figure 1C). Co-culture of SW- or SD-treated neutrophils with HLMEC increased P-selectin mRNA in HLMEC by 2.8- to 62-fold and 4- to 262-fold, respectively (Figure 1D), and increased P-selectin mRNA in neutrophils by 11- to 136-fold and 77- to 228-fold, respectively (Figure 1E).

### 2.2. Exposure of HLMEC to S-Proteins Induced P-Selectin Expression

**Figure 2:**
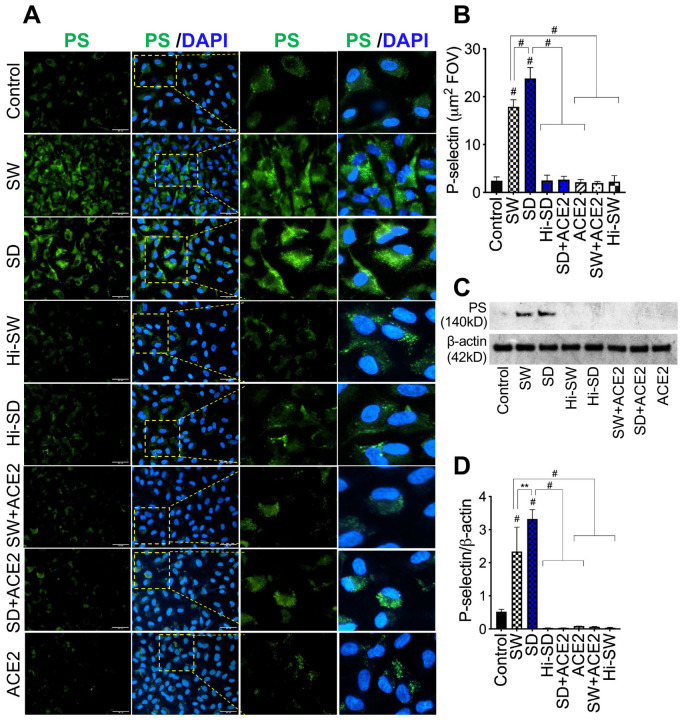
S-proteins induce P-selectin expression in HLMEC. HLMEC were treated with 1 nM S-proteins (SW or SD) for 12 h and P-selectin expression was quantified by immunofluorescence (**A**,**B**) and Western blot (**C**,**D**) analysis. DAPI (blue) was used for nuclear counterstaining. ImageJ software was used for densitometry quantification. For immunofluorescence images, five fields of view (FOV) were analyzed for each sample (**B**). For Western blot analysis, densitometry values were normalized to the sample’s β-actin levels (**D**). For panel (**A**), all images were at 20×. PS: P-selectin; control: untreated cells; ACE2: cells treated with rhACE2; Hi: cells treated with heat-inactivated S-proteins ** *p* = 0.01; # *p* < 0.0001.

Immunofluorescence imaging showed that, compared to controls, exposure (12 h) of HLMEC to SW or SD increased P-selectin expression by 7- to 9-fold and by 8.9- to 11.3-fold, respectively (Figure 2A,B). Western blot analysis further confirmed these findings; compared to controls, SW and SD, respectively, increased P-selectin levels by 4.5- to 63-fold and by 6.4- to 110-fold (Figure 2C,D).

### 2.3. Delta Variant S-Proteins Induced Higher P-Selectin Transcription and Expression Than the Wuhan Variant

At 12 h, compared to SW, SD induced significantly higher P-selectin mRNA in HLMEC following direct exposure (3.7-fold, Figure 1A) or co-culture with neutrophils (2- to 6.8-fold, Figure 1B); and induced higher P-selectin mRNA in neutrophils (7.6- to 12.4-fold, Figure 1C). For neutrophils treated with S-proteins and co-cultured with HLMEC, SD induced 4-fold higher P-selectin transcription in HLMEC at 24 h (Figure 1D) and 2- to 15-fold higher P-selectin mRNA in neutrophils (Figure 1E), compared to SW. Immunofluorescence and Western blot analyses also showed that SD increased P-selectin expression in HLMEC by 1.3- to 1.4-fold compared to SW (Figure 2). No significant increase in P-selectin transcription (Figure 1) or expression (Figure 2) was observed in cells treated with Hi-SW or Hi-SD; recombinant human (rh) ACE2 blocked or significantly abrogated SW- and SD-induced P-selectin.

### 2.4. Exposure of Human Neutrophils and HLMEC to S-Proteins and Neutrophil–Endothelial Interactions Induces Histone H3 Citrullination

**Figure 3:**
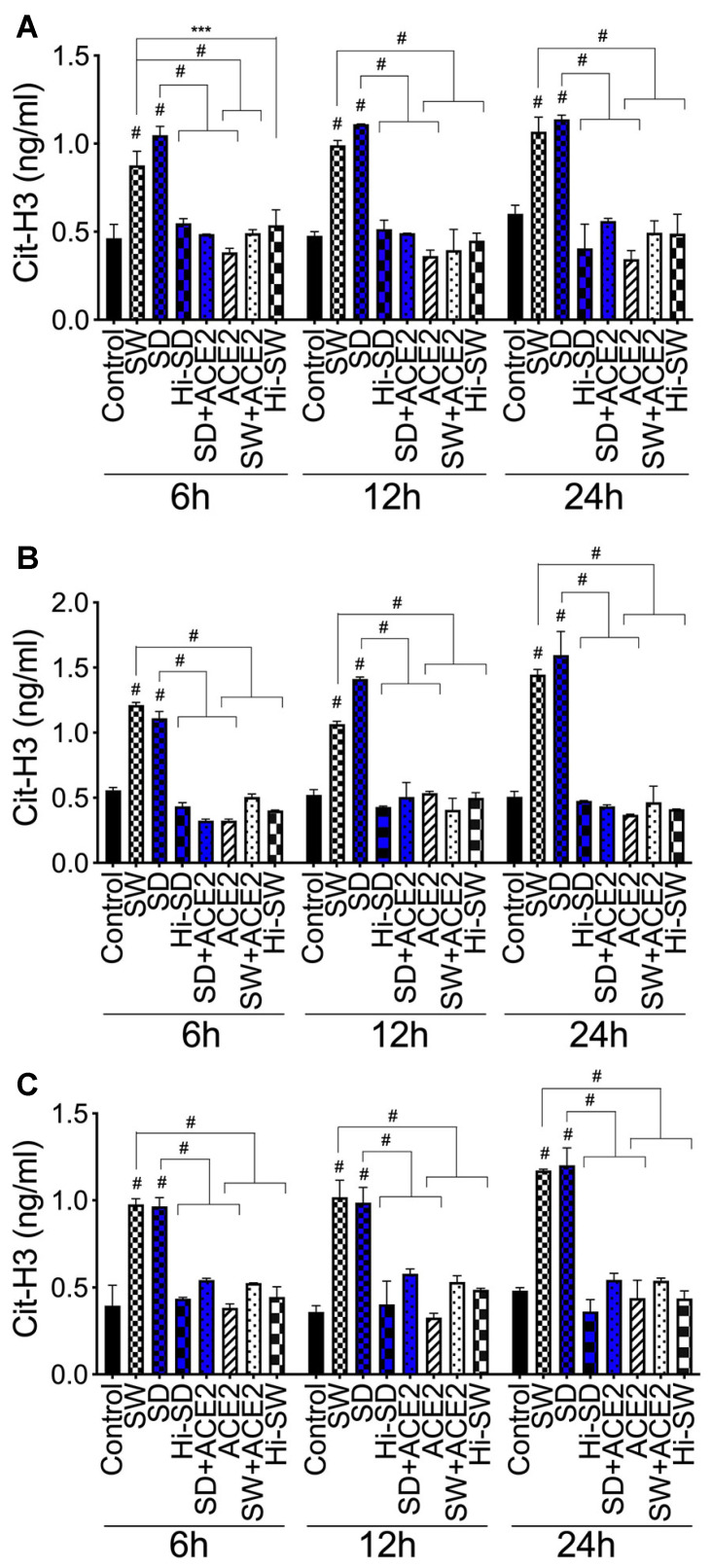
S-proteins and endothelial–neutrophil interactions induce/increase the expression and secretion of cit-H3. Human neutrophils were treated with 1 nM S-proteins (SW or SD) for 6–24 h (**A**). In separate experiments, neutrophils (**B**) and HLMEC (**C**) were treated with S-proteins for 6 h, washed, and co-cultured (for 6–24 h) with HLMEC (**B**) or neutrophils (**C**). Levels of cit-H3 in culture supernatants were quantified by ELISA. Data presented as mean ± standard deviation. Control: untreated cells; ACE2: cells treated with rhACE2; Hi: cells treated with heat-inactivated S-proteins. *** *p* = 0.0003; # *p* < 0.0001.

Hallmarks of NETosis include increased citrullination of histone proteins, including H3 [28,29,30]. Therefore, we assessed whether S-proteins and/or endothelial–neutrophil interactions affect the production of cit-H3. Compared to controls, SW and SD significantly increased cit-H3 levels in neutrophil culture supernatants following direct exposure (6–24 h) (1.6- to 3.3-fold, Figure 3A), co-culture of S-proteins-treated neutrophils with untreated HLMEC (2- to 4.3-fold, Figure 3B), or co-culture of S-proteins-treated endothelial cells with untreated neutrophils (1.7- to 3.3-fold, Figure 3C).

### 2.5. Exposure of HLMEC and Neutrophils to S-Proteins and Neutrophil–Endothelial Interactions Increased vWF Expression

**Figure 4:**
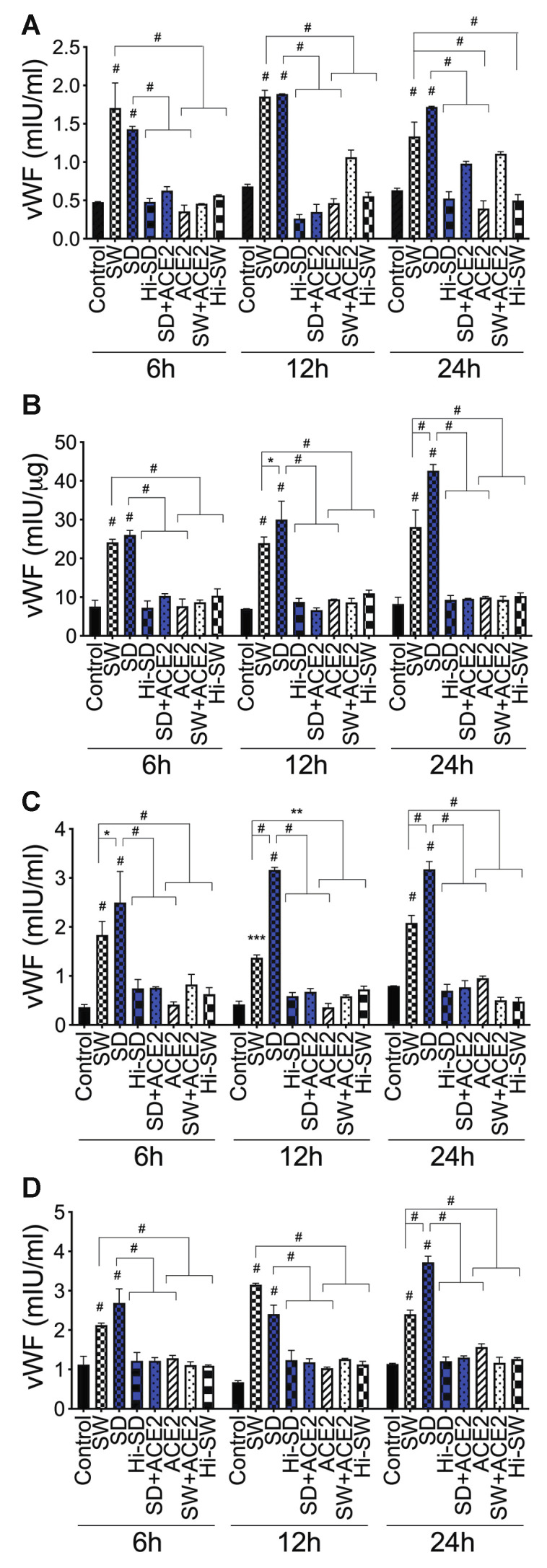
S-proteins and endothelial–neutrophil interactions induce vWF expression and release in HLMEC. (**A**,**B**) HLMEC were treated (for 6–24 h) with SW or SD (1 nM). HLMEC (**C**) and neutrophils (**D**) were treated (for 6 h) with SW or SD, washed, and co-cultured (for 6–24 h) with neutrophils (**C**) or endothelial cells (**D**). vWF levels in culture supernatants (**A**,**C**,**D**) and endothelial cell lysates (**B**) were quantified by ELISA. Data presented as mean ± standard deviation. Control: untreated cells; Hi: cells treated with heat-inactivated S-proteins; ACE2: cells treated with rhACE2. * *p* < 0.03; ** *p* < 0.01; *** *p* = 0.0002; # *p* < 0.0001.

Compared to controls, SW and SD significantly increased vWF levels in HLMEC culture supernatants (by 1.2- to 7.2-fold, Figure 4A) and cell lysates (2.2- to 5.2-fold, Figure 4B) following direct exposure (Figure 4A,B), co-culture of S-proteins-treated endothelial cells with untreated neutrophils (2- to 8.8-fold, Figure 4C), and co-culture of S-proteins-treated neutrophils with untreated HLMEC (1.5- to 4.6-fold, Figure 4D). Data showed a trend toward increased vWF following SD treatments and co-cultures, compared to SW (Figure 4).

### 2.6. Exposure of HLMEC and Neutrophils to S-Proteins and Endothelial-Neutrophil Interactions Increased IL-6 Expression

**Figure 5:**
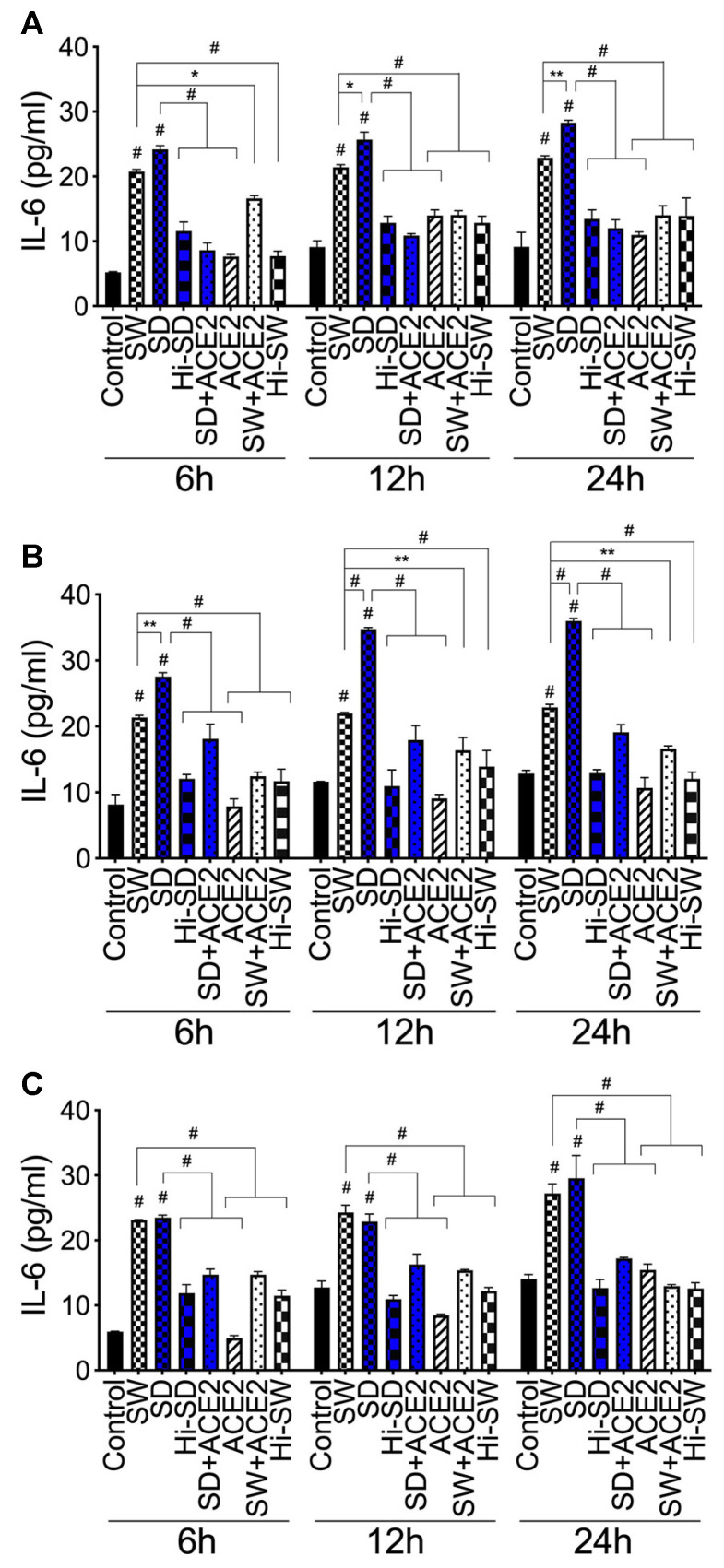
S-proteins and endothelial–neutrophil interactions increased IL-6 expression. (**A**) HLMEC treated (6–24 h) with SW or SD. HLMEC (**B**) and neutrophils (**C**) were treated (for 6 h) with SW or SD and co-cultured (for 6–24 h) with untreated neutrophils (**B**) or endothelial cells (**C**). IL-6 levels in culture supernatants were quantified by ELISA. Data presented as mean ± standard deviation. Control: untreated cells; ACE2: cells treated with rhACE2. Hi: cells treated with heat-inactivated S-proteins. * *p* < 0.02; ** *p* < 0.005; # *p* < 0.0001.

Compared to untreated cells, cells treated with Hi-SW, Hi-SD, or cells pretreated with rhACE2, exposure of HLMEC to SW or SD (6–24 h) increased IL-6 levels in culture supernatants by 1.2 to 4.6-fold (Figure 5A). Co-culture of SW- or SD-treated HLMEC with neutrophils increased IL-6 levels by 1.4 to 3.8-fold (Figure 5B). Compared to SW, SD induced significantly (1.3- to 1.6-fold) higher IL-6 expression following exposure to endothelial cells (Figure 5A) and co-culture of exposed endothelial cells with neutrophils (Figure 5B). Co-culture of SW- or SD-treated neutrophils with HLMEC increased IL-6 levels in culture supernatants by 1.4 to 4.7-fold (Figure 5C); rhACE2 blocked SW- and SD-induced IL-6. IL-6 levels in culture supernatants of cells treated with Hi-SW, Hi-SD, or rhACE2 were similar to untreated controls (Figure 5).

### 2.7. rTFPI Blocked S-Protein-Induced Citrullination of Histone H3, Expression and Secretion of vWF and IL-6

**Figure 6:**
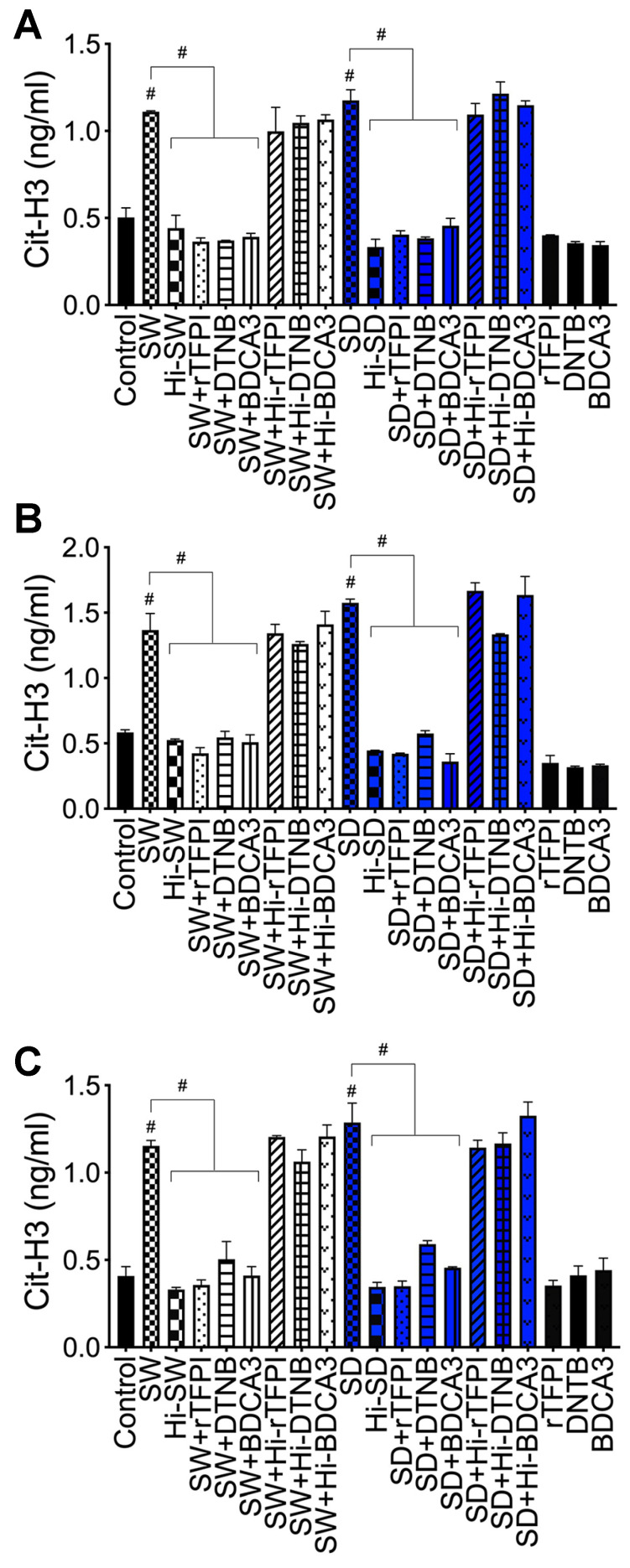
rTFPI, thrombomodulin (BDCA3), and thiol blockers (DTNB) prevent S-protein-induced H3 citrullination. (**A**): human neutrophils treated for 24 h with SW or SD, with or without rTFPI, DTNB, and BDCA3 (200 ng/mL). Neutrophils (**B**) and HLMEC (**C**) were treated (6 h) with SW or SD, with or without rTFPI, DTNB, and BDCA3, washed, and co-cultured (for 24 h) with HLMEC (**B**) or neutrophils (**C**). cit-H3 levels in culture supernatants quantified by ELISA. Data presented as mean ± standard deviation. Control: untreated cells; Hi: heat-inactivated (SW, SD, rTFPI, DTNB, BDCA3). # *p* < 0.0001.

H3 citrullination: Compared to controls, exposure (24 h) of neutrophils to SW or SD increased cit-H3 levels in culture supernatants by 2.2- to 3.5-fold (Figure 6A); co-culture of SW- and SD-treated neutrophils with HLMEC increased cit-H3 levels by 2.3- to 3.5-fold (Figure 6B), and co-culture of SW- and SD-treated HLMEC with neutrophils increased cit-H3 levels by 2.8- to 3.7-fold (Figure 6C). rTFPI blocked SW- and SD-induced H3 citrullination. Pretreatment with rTFPI reduced SW- and SD-induced H3 citrullination in neutrophils (by 3-fold, Figure 5A); reduced H3 citrullination induced by co-culture of SW- and SD-treated neutrophils with HLMEC (by 3.2- to 3.7-fold; Figure 6B); and reduced H3 citrullination induced by co-culture of SW- and SD-treated HLMEC with neutrophils (by 3.2- to 3.6-fold; Figure 6C).

**Figure 7:**
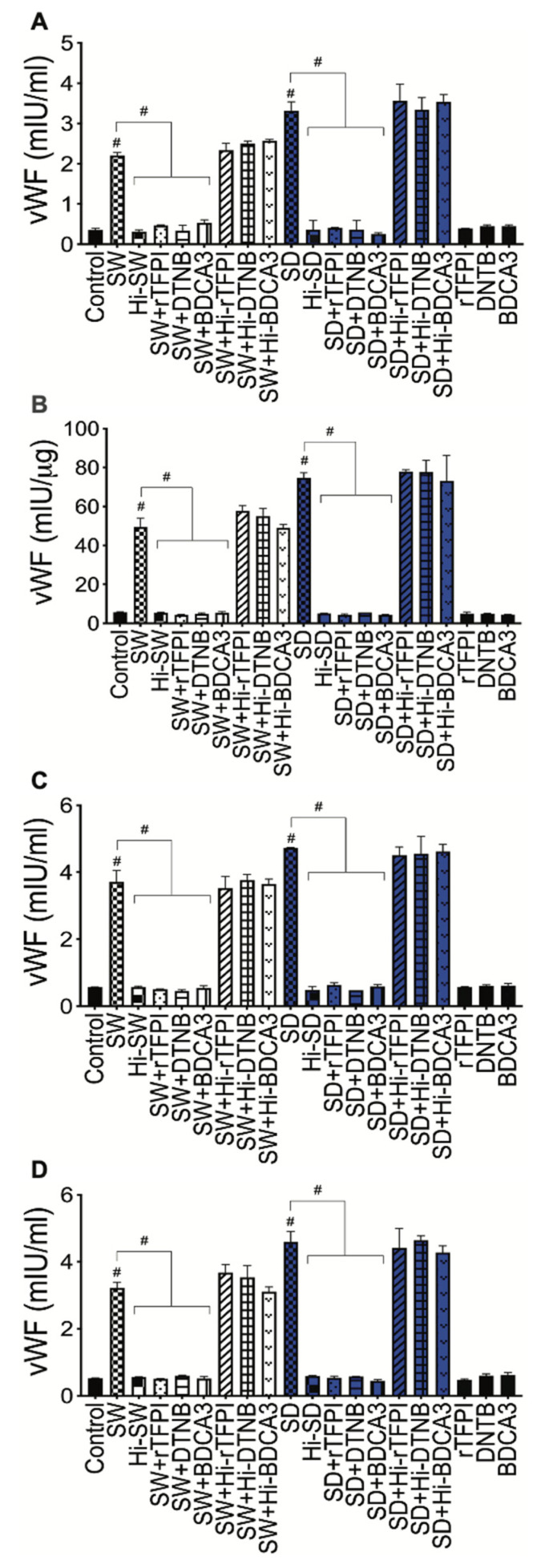
rTFPI, thrombomodulin, and thiol blockers prevent S-protein-induced vWF expression. (**A**,**B**) HLMEC were treated (24 h) with SW or SD, with or without rTFPI, DTNB, and BDCA3. HLMEC (**C**) and neutrophils (**D**) were treated (6 h) with SW or SD, with or without rTFPI, DTNB, and BDCA3, washed, and co-cultured (for 24 h) with neutrophils (**C**) or HLMEC (**D**). vWF levels in culture supernatants (**A**,**C**,**D**) and endothelial cell lysates (**B**) were quantified by ELISA. Data presented as mean ± standard deviation. Control: untreated cells; Hi: heat-inactivated (SW, SD, rTFPI, DTNB, BDCA3). # *p* < 0.0001.

vWF: Compared to controls, 24 h exposure of HLMEC to SW or SD increased vWF levels in culture supernatants (by 6.3- to 9.4-fold; Figure 7A) and cell lysates (by 8.7 to 14.6-fold; Figure 7B). Co-culture of SW- and SD-treated HLMEC with neutrophils increased vWF in culture supernatants by 6.5- to 9.8-fold (Figure 7C), and co-culture of SW- and SD-treated neutrophils with HLMEC increased vWF in culture supernatants by 5.8- to 8.7-fold (Figure 7D). rTFPI blocked SW- and SD-induced vWF expression and secretion. Pretreatment with rTFPI reduced SW- and SD-induced vWF in HLMEC culture supernatants (by 4.7- to 8-fold, Figure 7A) and cell lysates (by 11.4- to 17-fold, Figure 7B); reduced vWF induced by co-culture of SW- and SD-treated HLMEC with neutrophils (by 7.4-fold; Figure 7C); and reduced vWF induced by co-culture of SW- and SD-treated neutrophils with endothelial cells (by 6.3- to 8.5-fold; Figure 7D).

**Figure 8:**
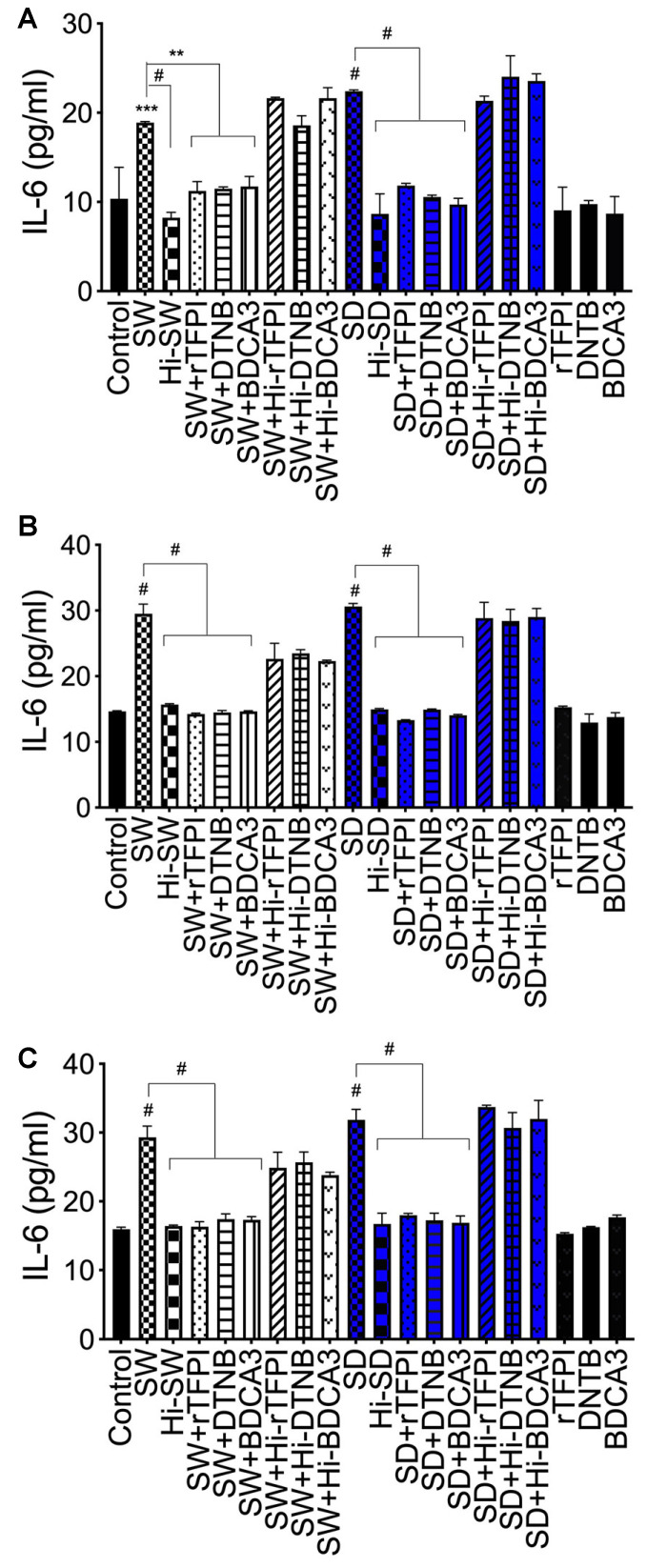
rTFPI, thrombomodulin, and thiol blockers prevent S-protein-induced IL-6 expression. (**A**) HLMEC were treated (24 h) with SW or SD, with or without rTFPI, DTNB, and BDCA3. HLMEC (**B**) and neutrophils (**C**) were treated (6 h) with SW or SD, with or without rTFPI, DTNB, and BDCA3, washed, and co-cultured (for 24 h) with neutrophils (**B**) or HLMEC (**C**). IL-6 levels in culture supernatants were quantified by ELISA. Data presented as mean ± standard deviation. Control: untreated cells; Hi: heat-inactivated (SW, SD, rTFPI, DTNB, BDCA3). ** *p* < 0.007; *** *p* = 0.0009; # *p* < 0.0001.

IL-6: Compared to controls, 24 h exposure of HLMEC to SW or SD increased IL-6 levels in culture supernatants by 1.8- to 2.6-fold (Figure 8A). Co-culture of SW- and SD-treated HLMEC with untreated neutrophils increased IL-6 expression by 2-fold (Figure 8B), and co-culture of SW- and SD-treated neutrophils with untreated HLMEC increased IL-6 expression by 1.8- to 2-fold (Figure 8C). rTFPI blocked SW- and SD-induced IL-6 expression. Pretreatment with rTFPI reduced SW- and SD-induced IL-6 in HLMEC culture supernatants (by 1.6 to 1.9-fold, Figure 8A); reduced IL-6 induced by co-culture of SW- and SD-treated HLMEC with neutrophils (by 2-fold; Figure 8B); and reduced IL-6 induced by co-culture of SW- and SD-treated neutrophils with endothelial cells (by 1.8-fold; Figure 8C).

### 2.8. DTNB Blocked S-Protein-Induced Citrullination of Histone H3, Expression and Secretion of vWF and IL-6

H3 citrullination. DTNB blocked SW- and SD-induced H3 citrullination. Pretreatment with DTNB reduced SW- and SD-induced H3 citrullination in neutrophils (by 3-fold, Figure 6A); reduced H3 citrullination induced by co-culture of SW- and SD-treated neutrophils with HLMEC (by 2.5- to 2.7-fold; Figure 6B); and reduced H3 citrullination induced by co-culture of SW- and SD-treated HLMEC with neutrophils (by 2.2- to 2.3-fold, Figure 6C).

vWF. DTNB blocked SW- and SD-induced vWF expression: reduced SW- and SD-induced vWF in HLMEC culture supernatants (by 6.5- to 9-fold, Figure 6A) and cell lysates (by 10- to 13-fold, Figure 7B); reduced vWF induced by co-culture of SW- and SD-treated HLMEC with neutrophils (by 8- to 10-fold; Figure 7C); and reduced vWF induced by co-culture of SW- and SD-treated neutrophils with EC (by 5- to 8-fold; Figure 7D).

IL-6. DTNB blocked SW- and SD-induced IL-6 expression and secretion: reduced SW- and SD-induced IL-6 in HLMEC culture supernatants (by 1.6- to 1.9-fold, Figure 8A); reduced IL-6 induced by co-culture of SW- and SD-treated HLMEC with neutrophils (by 2-fold; Figure 8B); and reduced IL-6 induced by co-culture of SW- and SD-treated neutrophils with HLMEC (by 1.7- 1.8-fold; Figure 8C).

### 2.9. Thrombomodulin Blocked S-Protein-Induced Citrullination of Histone H3 and Blocked vWF and IL-6 Expression and Secretion

H3 citrullination. Thrombomodulin (TM, BDCA3) blocked SW- and SD-induced H3 citrullination. Pretreatment with TM reduced SW- and SD-induced H3 citrullination in neutrophils (by 2.6- to 2.8-fold, Figure 6A); reduced H3 citrullination induced by co-culture of SW- and SD-treated neutrophils with HLMEC (by 2.7- to 4.4-fold; Figure 6B); and reduced H3 citrullination induced by co-culture of SW- and SD-treated HLMEC with neutrophils (by 2.8-fold; Figure 6C).

vWF. TM blocked SW- and SD-induced vWF expression: reduced SW- and SD-induced vWF in HLMEC culture supernatants (by 4- to 13-fold, Figure 6A) and cell lysates (by 9- to 17-fold, Figure 7B); reduced vWF induced by co-culture of SW- and SD-treated HLMEC with neutrophils (by 7- to 8-fold; Figure 7C); and reduced vWF induced by co-culture of SW- and SD-treated neutrophils with endothelial cells (by 6.2- to 10.2-fold; Figure 7D).

IL-6. TM blocked SW- and SD-induced IL-6 expression: reduced SW- and SD-induced IL-6 in HLMEC culture supernatants (by 1.6- to 2.3-fold, Figure 8A); reduced IL-6 induced by co-culture of SW- and SD-treated HLMEC with neutrophils (by 2-fold; Figure 8B); and reduced IL-6 induced by co-culture of SW- and SD-treated neutrophils with EC (by 1.7- to 1.9-fold; Figure 8C).

## 3. Discussion

The lungs are a prime target for SARS-CoV-2 infection. COVID-19 disease progression is often associated with acute respiratory distress syndrome involving severe lung injury, increased inflammation and coagulopathy [4,5,31,32], as well as increased leukocyte infiltration into tissues associated with endothelial apoptosis and microcirculatory clots [4,5,6]. The pathogenesis of these pulmonary complications in COVID-19 patients is unknown. Considering that neutrophils are the most abundant leukocytes in humans [33], that blood leukocytes infiltrate tissues by transmigrating through the vascular endothelium, and that S-proteins can be shed and are present in bodily fluids, microvessels, and tissues of SARS-CoV-2 infected patients [7,17], we investigated the effect of S-proteins and endothelial–neutrophils interactions on the adhesion molecule P-selectin, markers of endothelial injury and increased coagulopathy (vWF), NETosis (cit-H3) and inflammation (IL-6).

We demonstrate that exposure of HLMEC or neutrophils to S-proteins and endothelial–neutrophils interactions significantly increased IL-6 expression and secretion. S-proteins have also been shown to increase the production of IL-6 and other inflammatory cytokines and chemokines in endothelial cells from other vascular beds, including human aortic endothelial cells [34], as well as in human pulmonary epithelial cells [35,36], peripheral blood mononuclear cells and human and murine macrophages [37,38,39]. Studies of COVID-19 patients also showed significant increases in proinflammatory cytokines and chemokines, including IL-6, IL-1β, TNF-α, and granulocyte–macrophage colony-stimulating factor, in patients’ plasma, with much higher levels in the plasma of critically ill patients [40]. Our current study suggests that following SARS-CoV-2 infection, viral S-proteins contribute to inflammation of the lung endothelium and disease pathology; and that in the presence of S-proteins, endothelial–neutrophil interactions also induce inflammation.

P-selectin is expressed on activated endothelial cells, platelets and leukocytes, and functions as an adhesion molecule. During inflammatory responses, P-selectin plays a critical role in the recruitment and aggregation of platelets and leukocytes to the vascular wall and to areas of vascular and tissue injury [41,42]. Our data show that S-proteins increase P-selectin transcription and expression in HLMEC, and co-culture of S-protein-treated endothelial cells with non-treated neutrophils or co-culture of S-protein-treated neutrophils with non-treated endothelial cells further increases P-selectin in both HLMEC and neutrophils. These results suggest that S-protein-induced P-selectin would increase leukocyte adhesion to the lung endothelium and infiltration into lung tissues and that endothelial–neutrophil interactions further potentiate leukocyte adhesion and transmigration into tissues.

Our data also showed that exposure of HLMEC to S-proteins increased vWF expression and release, and co-culture of S-protein-treated endothelial cells with non-treated neutrophils, or co-culture of S-protein-treated neutrophils with non-treated endothelial cells, further increased vWF expression and secretion. vWF are stored in endothelial granules (Weibel–Palade bodies) and are released/secreted following endothelial activation [43]; vWF released further mediates the adhesion of platelets and leukocytes to the vascular endothelium and their recruitment to sites of injury. Thus, increased vWF release is a marker of endothelial activation and vascular injury [44]. vWF is a carrier of factor (F)-VIII and both vWF and F-VIII increase fibrin generation and coagulopathy [45]. Our previous study showed that exposure of human endothelial cells or neutrophils to S-proteins and endothelial–neutrophils interactions, increased expression and release of prothrombogenic factors, including tissue factor (TF), fibrinogen, and thrombin, via the TF pathway [46]. Our current findings are in agreement with clinical data showing that COVID-19 patients have significantly increased levels of circulating P-selectin, vWF antigen, and F-VIII activity [6,47,48,49]. The highest levels of P-selectin, vWF antigen, and F-VIII activity were observed among critically ill patients and were associated with thrombosis, severe disease, lower rates of hospital discharge, and higher mortality [6,47,48,49].

Our current study also demonstrates that exposure of neutrophils to S-proteins and neutrophil–endothelial interactions significantly increased the formation and release of cit-H3. Histone citrullination is a hallmark of NETosis and is mediated by peptidyl arginine deiminase-4 following neutrophil activation [28,50,51,52]. This citrullination leads to a loss of charge and deamination of histone arginine residues, which alter histone DNA and protein-binding properties and enable chromatin decondensation and the release of nuclear DNA fragments [50,51,52]. Thus, when activated, neutrophils can release NETs, which consist of web-like structures composed of double-stranded (ds) DNA, citrullinated histones and granule proteins [28,50,51,52]. NETosis has been linked to coagulopathy and thrombosis. NET components have been shown to degrade TFPI, thus activating the coagulation cascade TF pathway [53]. NETs released can further serve as a scaffold for the binding of other procoagulant molecules such as vWF, fibronectin, and fibrinogen [54], thus trapping circulating blood cells and promoting their aggregation, resulting in the formation of thrombi and vessel occlusion [54,55]. NET components (histones, dsDNA) further promote thrombosis by increasing the thickness, rigidity, and stability of fibrin fibers and impeding fibrinolysis [56,57].

Our current data showing that exposure of human neutrophils to S-proteins and neutrophil–endothelial interaction increases the production and release of a NET component (cit-H3) are in agreement with clinical studies showing increased NETosis in COVID-19. Plasma, neutrophils, and lung fluids from COVID-19 patients showed increased markers of neutrophil activation and NET components, including cit-H3, myeloperoxidase (MPO), and the MPO-DNA complex [19,40]. Exposure of healthy human neutrophils to SARS-CoV-2 virions also induced the release of NET components, including the MPO, MPO-DNA complex, and cit-H3 [19]. The NETs produced can further injure the vascular endothelium. There is evidence that NETs can induce toxicity, apoptosis, and dysfunction of the vascular endothelium; induce endothelial cell expression and release of adhesion molecules and TF, thus further promoting leukocyte recruitment to the vascular endothelium and thrombosis [53,58,59,60]. NETs produced following SARS-CoV-2 treatment of neutrophils also induced apoptosis in lung epithelial cells [19].

Our current data showed that compared to SW, exposure of HLMEC or neutrophils to SD and endothelial–neutrophil interactions induced 2- to 15-fold higher P-selectin transcription and significantly higher expression of P-selectin, IL-6 and vWF. Our previous studies also demonstrated that, compared to SW, exposure of HLMEC or neutrophils to SD and endothelial–neutrophil interactions induced significantly higher TF levels [46]. This evidence suggests that different SARS-CoV-2 genetic variants and subvariants that have been driving waves of infection and disease epidemiology since the beginning of the COVID-19 pandemic [61,62,63], can influence the production of pro-thrombotic factors, inflammation, leukocyte adhesion to the vascular endothelium and infiltration into tissues. These differential effects of SARS-CoV-2 variants and genotypes would influence disease pathology in infected individuals.

We previously demonstrated that exposure of HLMEC or neutrophils to S-proteins and neutrophil–endothelial interactions induced prothrombogenic factors (TF, F-V, thrombin, and fibrinogen), inhibited TFPI, and that both rTFPI and DTNB blocked S-protein-induced upregulation of F-V, thrombin, and fibrinogen [46]. TFPI is a serine protease inhibitor that inhibits TF activity and blocks the coagulation cascade extrinsic pathway [64,65,66]. Disulfide bonds are essential for TF activation and increased TF activity that drives the coagulation extrinsic pathway signaling cascade [67,68,69]. DTNB reacts with free thiol groups to prevent the formation of disulfide bonds [70], and thiol-based drugs can decrease S-proteins binding to ACE2, inhibit viral entry and infection, and decrease SARS-CoV-2-induced inflammation of lung neutrophils [71,72]. Our current data show that rTFPI and DTNB also blocked S-protein-induced expression and secretion of IL-6, vWF, and cit-H3. These results suggest that following direct contact of SARS-CoV-2 with the pulmonary endothelium or neutrophils, and endothelial–neutrophil interactions, viral S-proteins induce endothelial degranulation (leading to the release of vWF from cellular granules), NETosis and inflammation via the TF pathway and mechanisms involving functional thiol groups.

TM is an endothelial receptor and a natural anticoagulant that binds thrombin to form a stable thrombin–TM complex that induces fibrinolysis and prevent/reduce coagulation [27]. We previously demonstrated that TM blocked S-protein-induced upregulation of fibrinogen but had no effect on S-protein-induced expression of F-V or thrombin [46]. Our current study demonstrates that TM blocks S-protein-induced increases in IL-6, vWF, and cit-H3 production. These results suggest that SARS-CoV-2-induced vWF, NETosis, and inflammation occur downstream of the coagulation TF pathway, and as TM binds thrombin and limits the intrinsic and common pathways of the coagulation cascade, it abrogates vWF production, inflammation and NETosis.

Because S-proteins are shed by infected cells in vivo and most COVID-19 vaccines encode SARS-CoV-2 S-proteins, increases in markers of inflammation, coagulopathy, and NETosis following exposure of neutrophils and lung endothelial cells to S-proteins could explain some vascular complications observed in COVID-19 patients [73] and post-COVID-19 vaccination adverse events. In fact, reported post vaccination complications include increased vasculitis, endothelial activation, increased inflammatory cytokines and chemokines, and thrombosis [74,75,76]. Studies in a SARS-CoV-2 mouse model showed that the S-protein S1 subunit was primarily responsible for the observed lung injury, increase in inflammatory cytokines and blood cells in lung fluids [77]. In vitro studies also showed that S1 significantly decreased trans-endothelial electric resistance and increased endothelial permeability [77]. Our future studies will determine whether a specific S-protein subunit is responsible for the increased coagulopathy, histone citrullination and inflammation observed in our current study.

**Figure 9:**
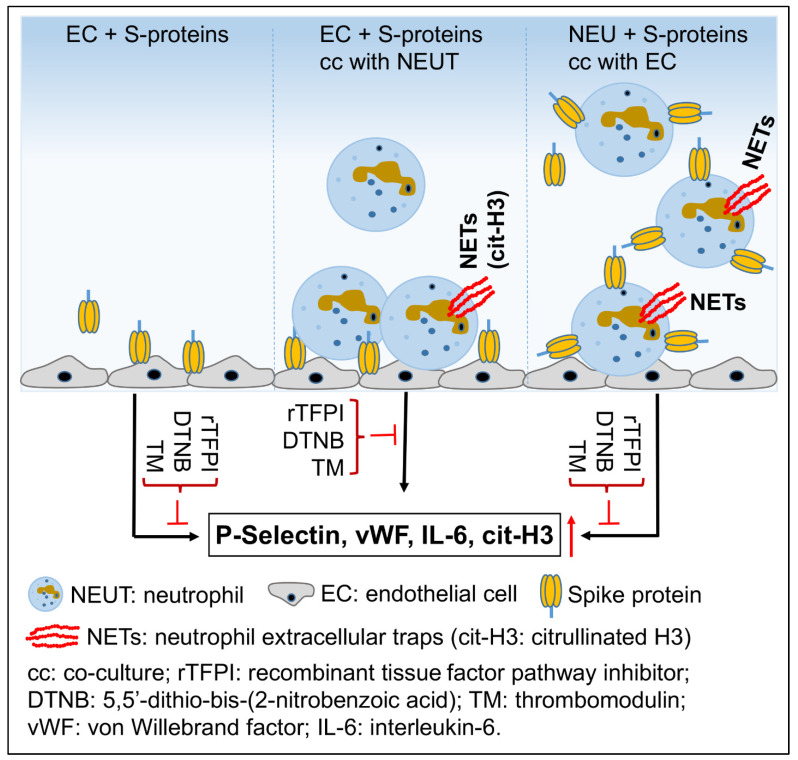
Model illustrating S-protein-induced P-selectin, vWF, IL-6 and cit-H3. Arrows indicate direct activation. The red arrows indicate upregulation; the red perpendicular symbol (**⊥**) indicates pharmacological inhibitors.

In summary, our current data demonstrate that exposure of primary HLMEC or neutrophils to S-proteins and endothelial–neutrophil interactions increased the transcription and expression of P-selectin (adhesion molecule), increased the expression and secretion of markers of endothelial activation and coagulopathy (vWF), NETosis (cit-H3) and inflammation (IL-6). rTFPI, DNTB, and TM prevented these S-protein-induced effects (Figure 9), which suggests that following SARS-CoV-2 contact with the lung endothelium or neutrophils and endothelial–neutrophil interactions, viral S-proteins induce inflammation, NETosis, and coagulopathy via the TF pathway and mechanisms involving free and functional thiol groups. These findings also suggest that therapeutic strategies against SARS-CoV-2-induced inflammation, NETosis, and coagulopathy could include supplementation with rTFPI, natural anticoagulants such as TM, and/or thiol-based drugs.

## 4. Materials and Methods

### 4.1. Reagents

Recombinant SARS-CoV-2 S-proteins, SW, SD, rhACE2, rTFPI, and TM (BDCA3), were purchased from R&D Systems (Minneapolis, MN, USA). DTNB was from Sigma-Aldrich (St. Louis, MO, USA). Anti-human CD66b and anti-human CD45 antibodies were from Stemcell Technologies (Cambridge, MA, USA); anti-human CD16 antibodies were from Ancell Corporation (Stillwater, MN, USA). Monoclonal P-selectin antibodies and DAPI were from Thermo Fisher/Invitrogen (Waltham, MA, USA), and β-actin antibodies were from Santa Cruz Biotechnology (Dallas, TX, USA).

### 4.2. HLMEC and Neutrophils

Primary HLMEC was obtained from Lonza (Houston, TX, USA), cultured to confluence as previously described [78,79,80], and used at passages 2 to 4. Blood samples were obtained from human donors seronegative for HIV-1, HIV-2, and hepatitis-B [80,81]. Neutrophils were isolated from fresh donor blood using the EasySep direct human neutrophil isolation kit (Stemcell Technologies), and their purity was confirmed by FACS as previously described using antibodies to human CD16, CD66b, and CD45 [46].

### 4.3. Cell Treatment and Endothelial–Neutrophil Co-Culture

S-proteins (both SW and SD) were used at 1 nM and rTFPI, DTNB, and BDCA3 at 200 ng/mL, based on previous studies showing that these doses do not decrease cellular viability [46,82]. Treatment of HLMEC and neutrophils with SW and SD, pre-treatment with rTFPI, DTNB, and BDCA3, culture, co-culture, collection of culture supernatants and harvesting of neutrophils and endothelial cells were performed as previously described [46]. Controls included untreated cells, cells treated with heat-inactivated (Hi) S-proteins, rTFPI, DTNB, or BDCA3, and cells pretreated with rhACE2 (1 μg/mL) to block S-protein binding.

### 4.4. RNA Isolation and Real-Time PCR

Total RNA was extracted from cells using the Trizol reagent (Life Technologies-Ambion, Austin, TX, USA), RNA quality was checked, and reverse transcription was performed using the Verso cDNA synthesis kit (ThermoFisher) as previously described [46]. Real-time PCR was performed using the LightCycler 480 II (Roche, Basel, Switzerland) Real-Time PCR System. Experimental details and cycling conditions were as previously described, using the following Applied Biosystems (Waltham, MA, USA) primers: Selectin-P (Hs00927900_m1) and GAPDH (Hs02786624_g1). P-selectin mRNA levels were quantified using the Delta-CT method and normalized to the sample’s GAPDH levels.

### 4.5. Human vWF, cit-H3, and IL-6 ELISA

Following treatments, culture supernatants and cells were collected; cells were lysed in mammalian cell lysis buffer (CelLytic M, Sigma) and their protein content quantified using the bicinchoninic acid assay, as previously described [83,84,85]. Levels of vWF, cit-H3, and IL-6 in each culture supernatant (100 μL), as well as vWF levels in cell lysates (100 μL containing 50 μg protein), were quantified by ELISA using human vWF (Abcam, Waltham, MA, USA), cit-H3 (Cayman Chemical, Ann Arbor, MI, USA), and IL-6 (Invitrogen) ELISA kits in accordance with the manufacturer’s protocols. Standard curves from human vWF, cit-H3, and IL-6 reference standards (provided with each kit) were used, respectively, to quantify vWF, cit-H3, and IL-6 levels in each sample. Data were analyzed using Student’s *t*-test (two-tailed) or analysis of variance, followed by Tukey’s multiple-comparison tests, as previously described [46].For all figures, data are presented as mean ± standard deviation.

### 4.6. Immunofluorescence Analysis

Primary HLMEC were cultured to confluence on collagen-coated coverslips, treated for 12 h with S-proteins and analyzed by immunofluorescence as previously described [85] using antibodies to P-selectin (Thermo Fisher Scientific, Waltham, MA, USA) diluted 1:100 in PBS containing 0.1% Tween 20 and 1% bovine serum albumin (PBST); and fluorescein isothiocyanate-conjugated secondary antibodies (1:2000 in PBST). Coverslips were mounted using Prolong^TM^ Gold anti-fade mounting medium with DAPI (Invitrogen) and sealed as we previously described. Images were captured using an Eclipse TE20000-U fluorescent microscope (Nikon, Melville, NY, USA) and an Infinity 3–6 urfm monochrome camera (Luminera, Lod, Israel). Semi-quantitative analysis of P-selectin expression was performed using computer-assisted image analysis of the ImageJ software, and five fields of view (FOV) were analyzed for each sample. The staining intensity was normalized to surface area (µm^2^) and averaged to estimate protein expression (µm^2^ FOV).

### 4.7. Western Blot Analysis

Primary HLMEC cultured to confluence in six-well plates were treated for 12 h with S-proteins, harvested and lysed in CelLytic^TM^ M buffer (Sigma) containing protease inhibitors. The total protein concentration in each sample was quantified using the Bicinchoninic Acid assay as previously described [83,85,86]. Protein samples (30 µg each) were analyzed by sodium dodecyl sulfate-polyacrylamide gel electrophoresis as previously described [83,85,86] using monoclonal antibodies to P-selectin and β-actin (each at 1:1000 dilution). Densitometry analysis was performed using the ImageJ (V.1.54f 29) software; each sample’s P-selectin level was normalized to its β-actin levels.

## Data Availability

All data generated or analyzed during this study are included in this publication and/or are available from the corresponding author on reasonable request.

## Abbreviations

DTNB	5:5′-dithio-bis-(2-nitrobenzoic acid)
TFPI	Tissue factor pathway inhibitor
rTFPI	Recombinant tissue factor pathway inhibitor
BDCA3/TM	Thrombomodulin
vWF	von Willebrand factor
IL-6	Interleukin-6
Cit-H3	Citrulinated histone 3
dsDNA	Double strand DNA
NETs	Neutrophils extracellular traps
MPO	Myeloperoxidase
TF	Tissue factor
F-V	Factor-V
F-VIII	Factor-VIII
SARS-CoV-2	Severe acute respiratory syndrome coronavirus-2
COVID-19	Coronavirus disease 2019
ACE2	Angiotensin-converting enzyme-2
rhACE2	Recombinant human ACE2
S-proteins	Spike proteins
SW	Spike protein: Wuhan variant
SD	Spike protein: delta variant
Hi	Heat-inactivated
ELISA	Enzyme-linked immunosorbent assay
PCR	Polymerase chain reaction
cDNA	Complementary DNA
GAPDH	Glyceraldehyde-3-Phosphate Dehydrogenase
